# A Single-Dose Intra-Articular Morphine plus Bupivacaine versus Morphine Alone following Knee Arthroscopy: A Systematic Review and Meta-Analysis

**DOI:** 10.1371/journal.pone.0140512

**Published:** 2015-10-16

**Authors:** Dong-xing Xie, Chao Zeng, Yi-lun Wang, Yu-sheng Li, Jie Wei, Hui Li, Tuo Yang, Tu-bao Yang, Guang-hua Lei

**Affiliations:** 1 Department of Orthopaedics, Xiangya Hospital, Central South University, Changsha, Hunan Province, China; 2 Department of epidemiology and health statistics, School of Public Health, Central South University, Changsha, Hunan Province, China; National Taiwan University, TAIWAN

## Abstract

**Objectives:**

The purpose of this study was to compare the efficacy and safety of a single-dose intra-articular morphine plus bupivacaine versus morphine alone in patients undergoing arthroscopic knee surgery.

**Methods:**

Randomized controlled trials comparing a combination of morphine and bupivacaine with morphine alone injected intra-articularly in the management of pain after knee arthrocopic surgery were retrieved (up to August 10, 2014) from MEDLINE, the Cochrane Library and Embase databases. The weighted mean difference (WMD), relative risk (RR) and their corresponding 95% confidence intervals (CIs) were calculated using RevMan statistical software.

**Results:**

Thirteen randomized controlled trials were included. Statistically significant differences were observed with regard to the VAS values during the immediate period (0-2h) (WMD -1.16; 95% CI -2.01 to -0.31; *p* = 0.007) and the time to first request for rescue analgesia (WMD = 2.05; 95% CI 0.19 to 3.92; p = 0.03). However, there was no significant difference in the VAS pain score during the early period (2-6h) (WMD -0.36; 95% CI -1.13 to 0.41; *p* = 0.35), the late period (6-48h) (WMD 0.11; 95% CI -0.40 to 0.63; *p* = 0.67), and the number of patients requiring supplementary analgesia (RR = 0.78; 95% CI 0.57 to 1.05; *p* = 0.10). In addition, systematic review showed that intra-articular morphine plus bupivacaine would not increase the incidence of adverse effects compared with morphine alone.

**Conclusion:**

The present study suggested that the administration of single-dose intra-articular morphine plus bupivacaine provided better pain relief during the immediate period (0-2h), and lengthened the time interval before the first request for analgesic rescue without increasing the short-term side effects when compared with morphine alone.

**Level of Evidence:**

Level I, meta-analysis of Level I studies.

## Introduction

Knee arthroscopy is a kind of surgical procedure which can minimize soft tissue damage and is commonly performed by orthopedists on a day-case basis. However, a variable amount of pain is often accompanied with this procedure. Previous studies have reported that the incidence of moderate-to-severe pain following knee arthroscopy were nearly 70% [1,2]. Inadequate control of postoperative pain may delay recovery and result in prolonged hospitalization and increasing medical care costs [3,4]. Therefore, various investigations have been conducted in an attempt to search an ideal analgesic technique that can provide satisfactory analgesia and with therapeutic safety. Intra-articular (IA) injection of local anaesthetics and analgesics has become increasingly popular, for it is a simple and practicable method that can well adapt to the characteristic of arthroscopy [5]. Though many proposals including bupivacaine, morphine, lidocaine, fentanyl, tramadol, ketorolac, ropivacaine, hyaluronic acid and dexamethasone have been made since the technique was described in randomized controlled trial (RCT) in 1989 [6], no consensus about the most effective drug was reached.

Morphine, an opioid, and bupivacaine, a local anaesthetic, are often used intra-articularly for the postoperative pain management. Both of their analgesic efficacy and safety have been conformed in the previous meta-analysis of our group [7,8]. However, the mechanism of these two substances are different. Several studies showed that effective analgesia following knee arthroscopy can be provided with IA bupivacaine, but the peak blood concentration was produced within the first hour and the duration of analgesia is only 2–4 h, or even 1–2 h [6,9–11]. In contrast, morphine injected intra-articularly can provide a later onset of peak action at 3–6 h, and the pain relief was sought up to 24 h, or even 48h [12–15]. Thus, there is a possibility that an earlier onset and longer duration of analgesia might be obtained by injecting a combination of these two drugs into the knee joint space. During the past two decades, some studies were conducted advocating IA morphine plus bupivacaine to enhance analgesia in patients undergoing knee arthroscopy, and satisfactory effect were obtained compared with placebo or bupivacaine [16–20]. However, the analgesic benefit of IA a combination of morphine and bupivacaine remains controversial when compared with morphine alone. Allen et al showed that 1mg morphine combined with 0.25% bupivacacine could resulted in better pain relief than 1 or 2 mg morphine alone for the first 6 hours [21]. Similar results were seen in some other studies, and the superior analgesia provided by a combination of morphine and bupivacaine were observed in different periods, ranging from first 1 hour, to first 2 and 4 hours [22–26]. Nevertheless, there were also some studies failing to show any better analgesic effect of IA morphine plus bupivacaine when compared with morphine alone [16,27]. With accumulated evidences, our goal was to evaluate the efficacy and safety of IA morphine plus bupivacaine versus morphine alone in patients following knee arthroscopy by conducting a systematic review and meta-analysis. We hypothesized that IA morphine combined with bupivacaine could provide better pain relief than morphine without increasing the adverse effects.

## Materials and Methods

### Electronic searches

This systematic review and meta-analysis was conducted according to the Preferred Reporting Items for Systematic Reviews and Meta-analysis statement. We searched the electronic sources including MEDLINE/PubMed, the Cochrane Central Register Trials (CENTRAL) and Embase databases to identify RCTs that compared IA a combination of morphine and bupivacaine with morphine alone in patients undergoing arthroscopic surgery up to August 10, 2014. The search terms were as follows: “arthroscopy”, “arthroscopic”, “arthroscope”, “anterior cruciate ligament”, “bupivacaine”, “morphine”, “randomized controlled trial”. No restrictions were applied (S1 File). Meanwhile, citation lists from retrieved articles and recent reviews were searched.

### Study selection

Two independent reviewers assessed the obtained articles. Studies were considered to be eligible if they met the following conditions: (1) vivo studies using human subjects following arthroscopy, (2) randomized controlled studies, (3) experimental group receiving a combination of IA morphine and bupivacaine, (4) participants in control group injected with morphine only, (5) both experimental and control groups did not add other analgesics, (6) English literature only. We excluded the articles based on the following criteria: (1) vitro studies, animal studies, reviews, letters, case reports and non-randomized controlled studies, (2) arthroscope-assisted surgeries were not performed in the knee joint, (3) experimental or control group received additional analgesics, (4) non-English literature. We performed a consensus procedure for study selection. If consensus was not reached, a third reviewer would make a judgment.

### Data extraction and quality assessment

Two authors independently extracted the basic information and outcomes of the included 13 studies through a standardized form. Study characteristics that we retained included the first author, origin, mean age, sex ratio, number of patients in each group, volume of injected fluid, concentration of bupivacaine and doses of morphine in the combined groups, doses of morphine in morphine groups, follow-up time point, type of anesthesia, use epinephrine or not, type of surgery and the time of IA injection. Postoperative pain intensity measured on a visual analogue scale (VAS) was chosen as primary outcome in this systematic review and meta-analysis. The VAS data will be divided 10 to get a uniform scale from 1 to 10, if it ranged from 0 to 100. Secondary outcome measures included time to first rescue medication, number of patients requiring supplementary analgesics and adverse reactions.

Modified oxford scale (MOS) was used to evaluate the methodological quality of included studies by two independent researchers [28]. The MOS classifies the randomized controlled trials according to their randomized method, concealment allocation, blinding and reporting of participant withdraws through a 0-to-7-point interval. A total score greater or equal to 4 was considered to be a high quality study, otherwise, a low quality study. In order to reach a more objective result, the information including the name of the journal, the names of the authors, institutions and origin were concealed to the reviewers. Besides, any of disagreement was to be discussed and a third reviewer was to be consulted if necessary.

### Statistical analysis

Review Manager 5.2 software (RevMan 5.2, The Cochrane Collaboration, Oxford, UK) was used for data analysis. To continuous outcome measures, postoperative pain intensity reporting on a visual analogue scale (VAS) and time to first rescue analgesics, Weighted mean difference (SMD) was calculated with corresponding 95% confidence intervals (95% CI). Dichotomous data, number of patients requiring supplementary analgesics, was displayed as relative risks (RR) and its 95% confidence intervals. Standard deviation (SD) of outcome would be estimated according to the sample size, the standard error (SE) or 95% confidence interval (95%CI) if it was not presented. The means and SDs would be manually measured if they were provided by figures. If there were two morphine groups with different doses in one study, only the group using the same dose as the combined group was selected [21,23,25,29]. Postoperative pain intensity were analyzed in three different periods: immediate (0-2h), early (2-6h), late (6-48h) [12], and VAS pain score at the last follow-up time point of the three periods were extracted respectively. Studies that reported the mean of pain score solely and the median of pain will be used for qualitative meta-analysis.

To assess the heterogeneity among the included studies, *Q* and *I* ^*2*^ statistics were calculated, with a *p* value >0.05 of the *Q* statistics and *I* ^*2*^ value <50% indicating statistical homogeneity. If the studies were statistically homogeneous, a fixed effect model was used to conduct a meta-analysis; otherwise, the random model was applied. Besides, sensitive analyses were conducted to evaluate the influence of different exclusion criteria on overall effect size. For the assessment of publication bias, Begg’s tests (*p* ≤ 0.05 indicating statistically significant) were used and funnel plots were inspected [30]. These statistical process were performed with the Review Manager 5.2 software (RevMan 5.2, The Cochrane Collaboration, Oxford, UK) and STATA version 11.0 (StataCorp LP, College Station, Texas, USA).

## Results

### Characteristics of included studies

**Fig 1:**
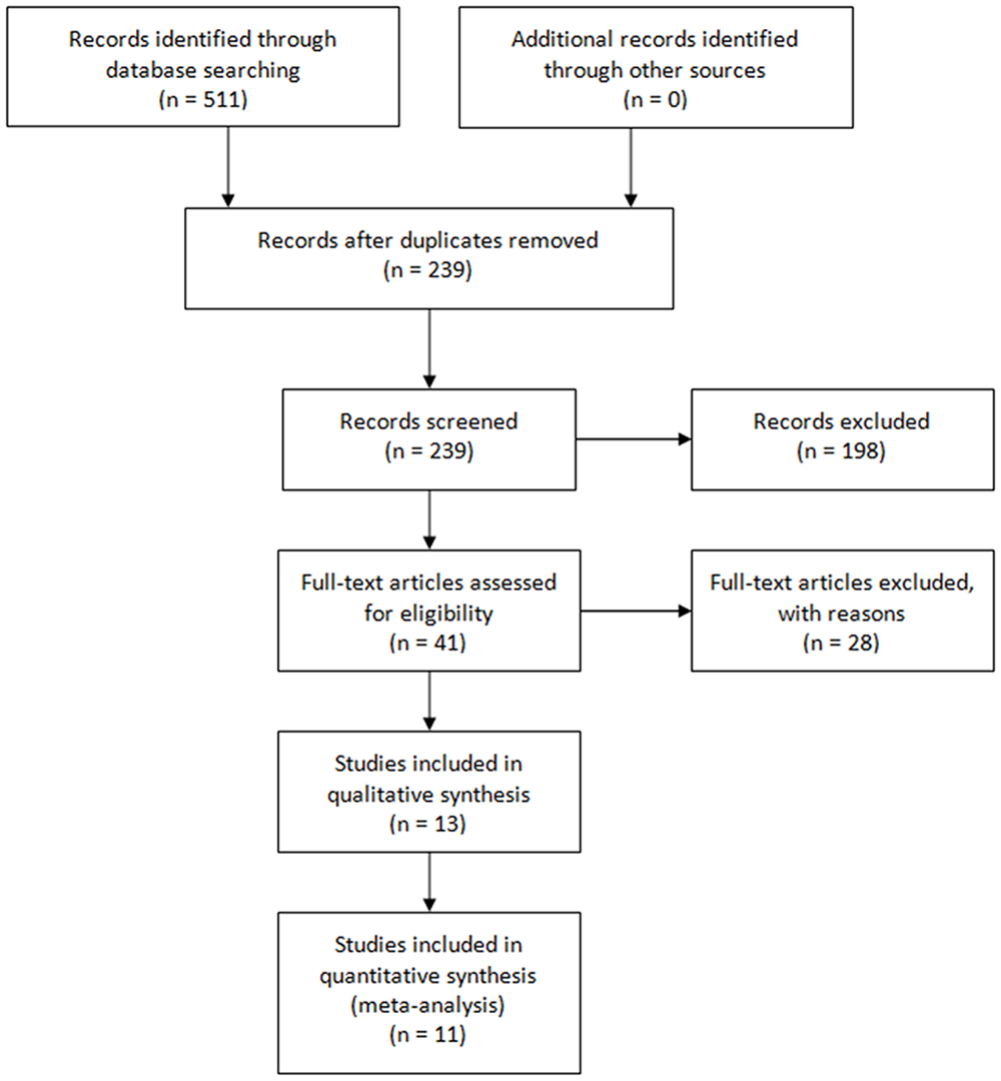
Flow chart of the selection of articles.

A total of 239 articles were initially identified from the electronic database. Finally, forty one articles were selected for reading full text, with 13 studies meeting the inclusion criteria (Fig 1). Study, participants and intervention characteristics of the 13 trials were summarized in Table 1. The studies originated from the United States of America (n = 7), Sweden (n = 2), Singapore (n = 1), Spain (n = 1), Italy (n = 1), and the United Kingdom (n = 1), involving 564 participants.

**Table 1:** Characteristics of the included studies. N, number of patients per group; B, bupivacaine; M, morphine; h, hour; MOS, modified oxford scale; NA, not available; ACL, anterior cruciate ligament, USA, United States of America, UK, United Kingdom.

Study	Origin	Age	Sex(Male/Female)	n(B+M/ M)	Injection Volume	Concentration (B) and Doses(M)	Doses (M)	Time (h)	Type of anesthesia	Epinep-hrine	Type of surgery	Intra-articular injection time	MOS
Aasbo V	USA	39.5	29/24	27/26	20ml	0.25%+3mg	3mg	1,2,3,4,8,12,24,72,168	General	No	Elective, diagnostic knee arthroscopy	At the completion of surgery	4
Allen GC	USA	36.9	46/14	30/30	30ml	0.25%+1mg	1mg	1,2,3,4,5,6,24	General	Yes	Diagnostic arthroscopy, meniscectomy, removal of loose body	3–5 min before releasing the tourniquet	6
Bjornsson A	Sweden	32.9	33/7	19/21	20ml	0.25%+1mg	1mg	0.5,1,1.5,2,8,24,48	General	No	Arthroscopic knee surgery	5–10 min before releasing the tourniquet	4
Chan ST	Singapore	24.5	20/0	10/10	20ml	0.25%+1mg	1mg	1,2,4,24	General	No	Therapeutic arthroscopic knee surgery	Before the arthroscope was removed	4
De Andres J	Spain	35.3	28/25	25/27	20ml	0.25%+1mg	1mg	0.33,4,10,16,24	General	No	Elective arthroscopic meniscectomy	At the completion of surgery	5
Denti M 1	Italy	38.4	NA	11/11	20ml	0.5%+2mg	2mg	1,3,6,12,24	Spinal	No	Diagnostic arthroscopy, arthroscopic surgery	10 min before releasing the tourniquet	6
Denti M 2	Italy	38.4	NA	10/10	20ml	0.5%+2mg	2mg	1,3,6,12,24	General	No	Diagnostic arthroscopy, arthroscopic surgery	10 min before releasing the tourniquet	6
Joshi GP	USA	31.6	15/5	10/10	25ml	0.25%+5mg	5mg	1,2,4,8,24	General	No	Arthroscopy for diagnostic purpose or meniscectomy	At the completion of surgery	3
Karlsson J	Sweden	NA	NA	10/10	20ml	0.375%+1mg	1mg	2,4,6, 24,48	General	No	ACL reconstruction	At the completion of surgery	6
Khoury GF	USA	44.5	NA	11/11	20ml	0.25%+1mg	1mg	1,2,3,4,24,48	General	No	Diagnostic tissue excisions, partial or total meniscectomies, and lateral release	Before the arthroscope was removed	3
McSwiney MM	UK	32.6	17/3	10/10	25ml	0.25%+5mg	5mg	0,0.5,1,1.5,2,4,8,12,24	General	No	Therapeutic meniscectomy	At the completion of surgery	4
Reuben SS	USA	27	NA	25/25	30ml	0.25%+5mg	5mg	1,2,24	General	No	ACL reconstruction	At the completion of surgery	4
Ruwe PA	USA	43.8	37/11	22/26	20ml	0.5%+2mg	2mg	0.5,1, 24,48	General	No	Diagnostic arthroscopy, partial meniscectomy, debridement, loose body removal, and lateral release	At the completion of surgery	5
Tetzlaff JE	USA	NA	NA	12/9	60ml	0.25%+1mg	1mg	0.5,1, 1.5,2,4	General	No	ACL reconstruction	At least 20min before incisions	6

### Pain measures

**Fig 2:**
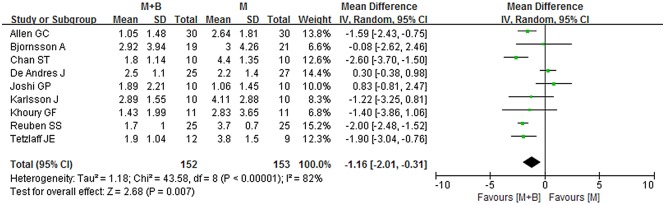
Forest plot of meta-analysis: visual analogue scale score of postoperative pain intensity in the immediate period (0-2h). M, morphine; B, bupivacaine; SD, standard deviation; IV, inverse variance; CI, confidence interval.

**Fig 3:**
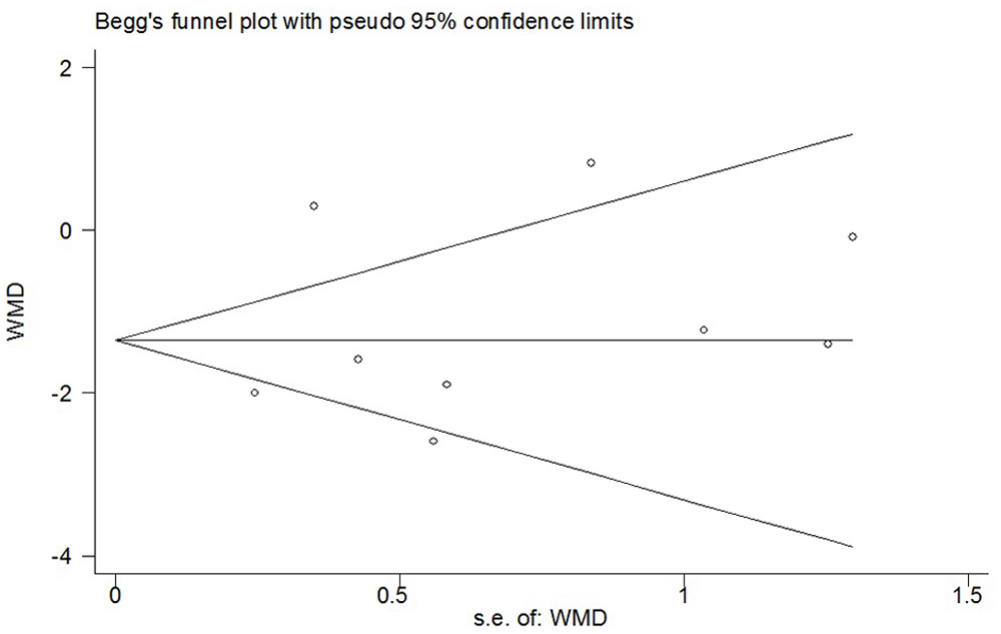
Funnel plot of meta-analysis: visual analogue scale score of postoperative pain intensity in the immediate period (0-2h). WMD, weighted mean difference.

During the immediate period (0-2h), data provided by nine studies were pooled [5,13,16,21,22,24–26,31]. Overall, the combined group showed a statistically significant lower postoperative pain intensity compared with morphine group (WMD -1.16; 95% CI -2.01 to -0.31; *p* = 0.007), while significant heterogeneity was observed (*I* ^*2*^ = 82%; *p*<0.00001) (Fig 2). Sensitivity analyses showed that the results were stable and reliable. The overall WMD did not change substantially when studies of poor methodological quality were excluded or other exclusions were applied; it ranged from -1.38 (95% CI -2.29 to -0.48; *p* = 0.003) to -1.05 (95% CI -2.00 to -0.10; *p* = 0.03) (Table 2). Substantial asymmetry was not identified in the funnel plot (Begg’s test, *p* = 0.348) (Fig 3). Of the other three studies whose data were not available for quantitative meta-analysis, two showed negative results and one showed positive result [23,27,32].

**Table 2:** Results of sensitivity analyses.

Exclusion of studies	pooled results of the remaining studies		Heterogeneity of the remaining studies	
	WMD/RR	*p*	*I* ^*2*^	*p*
VAS values(0-2h)				
Poor methodological quality	-1.38	0.003	84%	<0.000001
Mixed with epinephrine	-1.08	0.03	84%	<0.000001
Injection time (before incisions)	-1.05	0.03	84%	<0.000001
Small sample size (<10 in groups)	-1.05	0.03	84%	<0.000001
VAS values(2-6h)				
Poor methodological quality	-0.44	0.37	74%	0.002
Mixed with epinephrine	-0.16	0.71	63%	0.01
Injection time (before incisions)	-0.25	0.58	71%	0.002
Spinal anesthesia	-0.54	0.15	64%	0.01
Small sample size (<10 in groups)	-0.25	0.58	71%	0.002
VAS values(6-48h)				
Poor methodological quality	0.41	0.39	71%	0.004
Mixed with epinephrine	0.22	0.56	62%	0.02
Spinal anesthesia	0.11	0.67	37%	0.14
Time to first request for rescue				
Analgesia				
Mixed with epinephrine	2.2	0.05	97%	<0.00001
Number of patients requiring supplementary analgesia				
Poor methodological quality	0.77	0.09	0%	0.52
Mixed with epinephrine	0.87	0.45	0%	0.88

**Fig 4:**
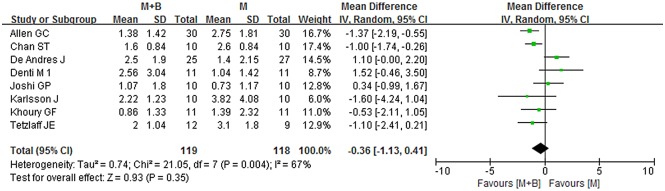
Forest plot of meta-analysis: visual analogue scale score of postoperative pain intensity in the early period (2-6h). M, morphine; B, bupivacaine; SD, standard deviation; IV, inverse variance; CI, confidence interval.

**Fig 5:**
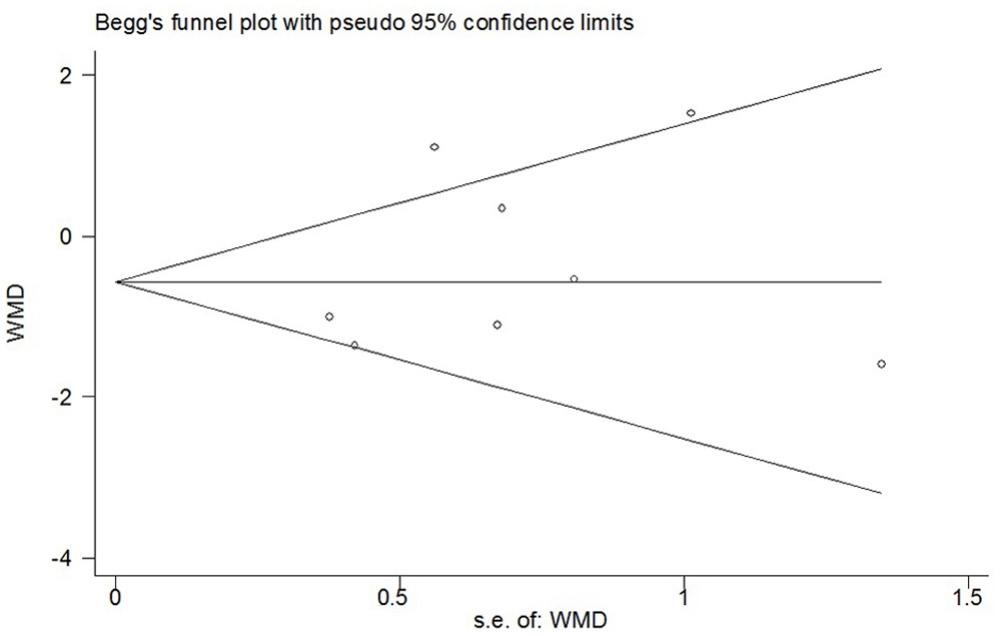
Funnel plot of meta-analysis: visual analogue scale score of postoperative pain intensity in the early period (2-6h). WMD, weighted mean difference.

During the early period (2-6h), data provided by eight studies were pooled [5,13,16,21,22,25,26,29]. Overall, no statistically significant difference was observed between the combined group and morphine group (WMD -0.36; 95% CI -1.13 to 0.41; *p* = 0.35), with significant heterogeneity being observed (*I* ^*2*^ = 67%; *p* = 0.004) (Fig 4). Sensitivity analyses suggested that the results were relatively stable and reliable. Exclusion of studies of poor methodological quality or other exclusions did not materially alter the overall combined WMD, with a range from -0.54 (95% CI -1.29 to 0.20; *p* = 0.15) to -0.16 (95% CI -1.01 to 0.68; *p* = 0.71) (Table 2). Substantial asymmetry was not identified in the funnel plot (Begg’s test, *p* = 0.386) (Fig 5). The other three studies for systematic review also showed no significant difference between the combined group and morphine group.

**Fig 6:**
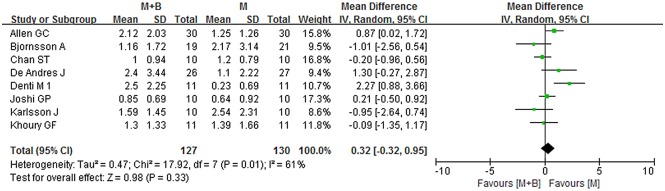
Forest plot of meta-analysis: visual analogue scale score of postoperative pain intensity in the late period (6-48h). M, morphine; B, bupivacaine; SD, standard deviation; IV, inverse variance; CI, confidence interval.

**Fig 7:**
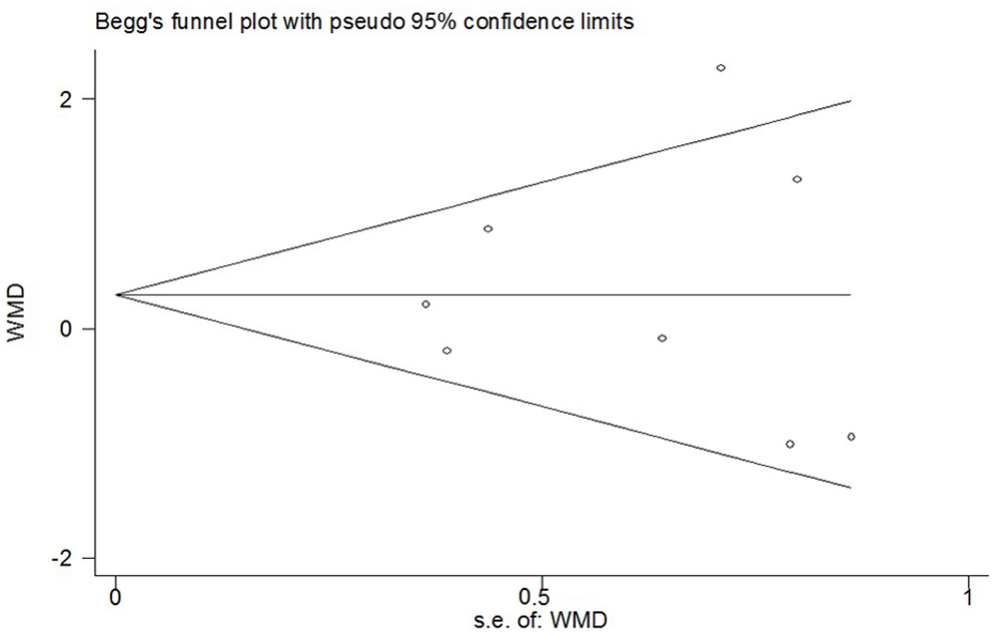
Funnel plot of meta-analysis: visual analogue scale score of postoperative pain intensity in the late period (6-48h). WMD, weighted mean difference.

During the late period (6-48h), data provided by eight studies were pooled [5,13,16,21,22,26,29,31]. Overall, no statistically significant difference was observed between the combined group and morphine group (WMD 0.32; 95% CI -0.32 to 0.95; *p* = 0.33), but significant heterogeneity was observed (*I* ^*2*^ = 61%; *p* = 0.01) (Fig 6). Sensitivity analyses were conducted, and a summary of the results was presented in Table 2. By omitting studies of poor methodological quality or other exclusions being applied, the overall WMD did not change substantially and it ranged from 0.11 (95% CI -0.40 to 0.63; *p* = 0.67) to 0.41 (95% CI -0.51 to 1.33; *p* = 0.39). *I* ^*2*^ statistic declined to 37% when the studies whose participants receiving spinal anesthesia were removed (WMD 0.11; 95% CI -0.40 to 0.63; *p* = 0.67). Substantial asymmetry was not identified in the funnel plot (Begg’s test, *p* = 0.711) (Fig 7). Besides, no significant difference was observed in the systematic review.

### Time to first analgesic request

**Fig 8:**
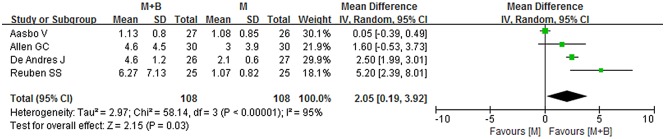
Forest plot of meta-analysis: time to first analgesic request. M, morphine; B, bupivacaine; SD, standard deviation; IV, inverse variance; CI, confidence interval.

**Fig 9:**
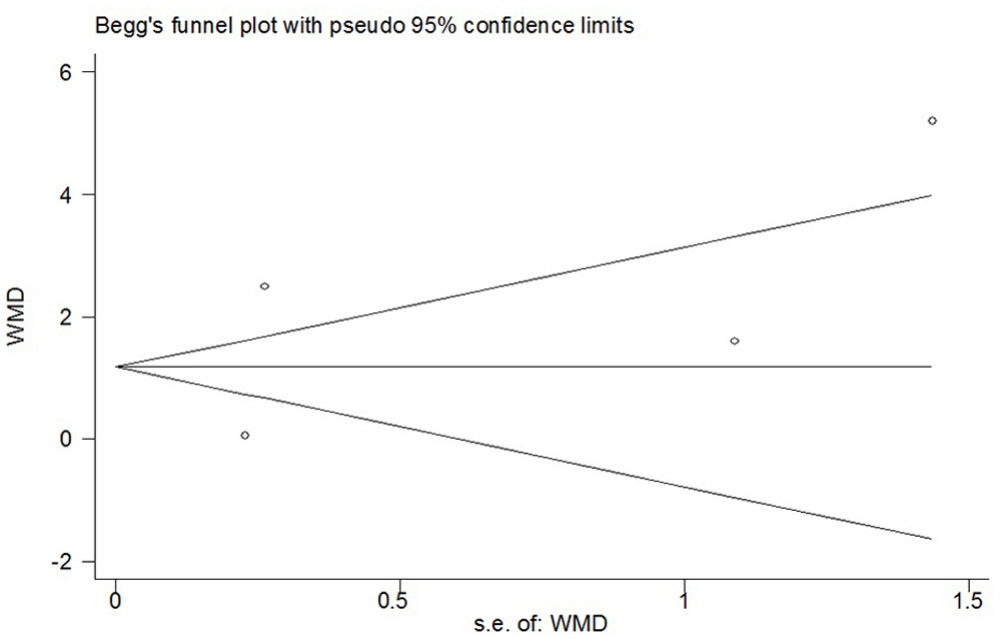
Funnel plot of meta-analysis: time to first analgesic request. WMD, weighted mean difference.

A total of four studies provided data on time to first analgesic request [5,21,24,27]. The pooled data suggested a significant difference in time to first analgesic request (WMD = 2.05; 95% CI 0.19 to 3.92; p = 0.03), with a significant heterogeneity (*I* ^*2*^ = 95%; *p <* 0.00001) (Fig 8). Sensitivity analyses showed that the results were stable and reliable (Table 2). By excluding studies in which experimental groups mixed with epinephrine, the overall WMD was 2.20 (95% CI 0.01 to 4.40; *p* = 0.05) and did not change substantially (Table 2). Funnel plot did not identify substantial asymmetry, and Begg’s test was conducted (*p* = 0.734) (Fig 9).

### Number of patients requiring supplementary analgesia

**Fig 10:**
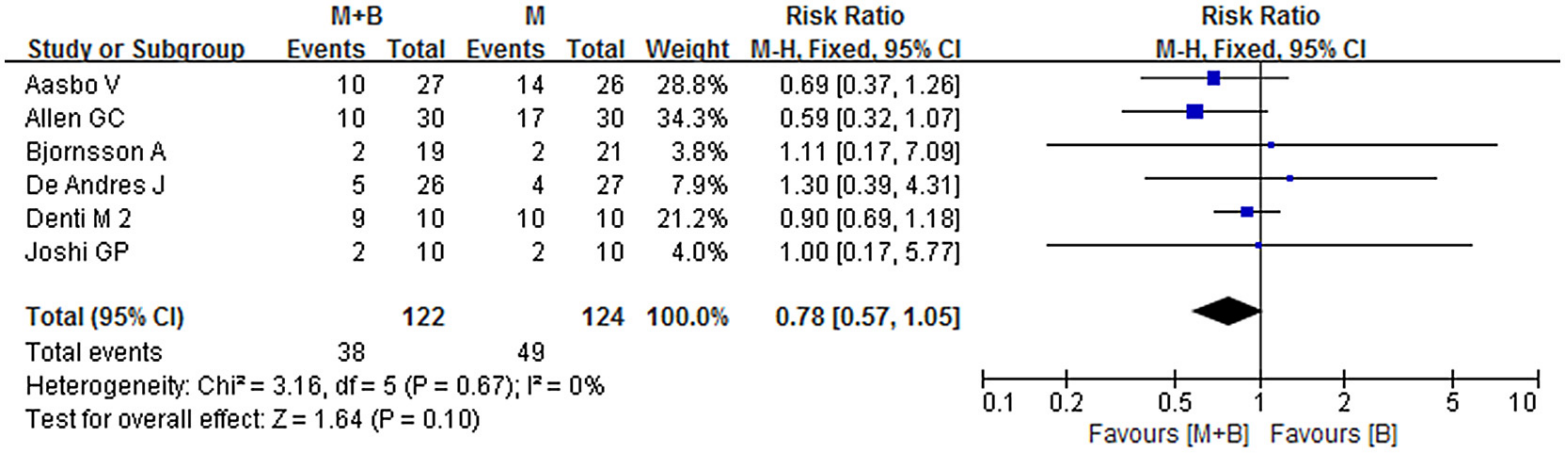
Forest plot of meta-analysis: number of patients requiring supplementary analgesia. M, morphine; B, bupivacaine; SD, standard deviation; IV, inverse variance; CI, confidence interval.

**Fig 11:**
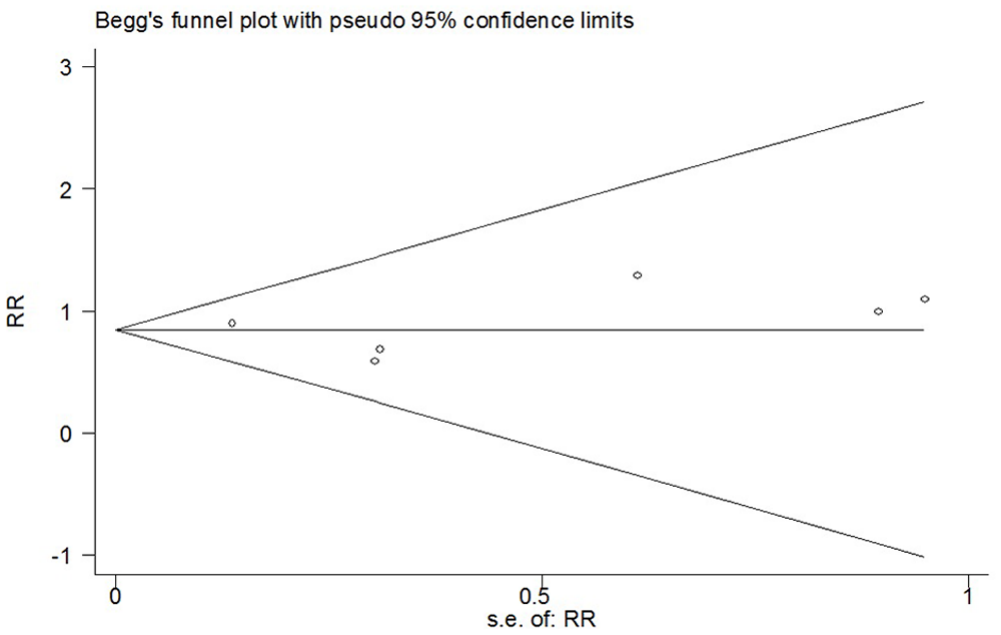
Funnel plot of meta-analysis: number of patients requiring supplementary analgesia. WMD, weighted mean difference.

A total of six studies provided data on the number of patients requiring supplementary analgesia [5,16,21,27,29,31]. No statistical significant difference was observed between experimental and control groups (RR = 0.78; 95% CI 0.57 to 1.05; *p* = 0.10), with a substantial heterogeneity (*I* ^*2*^ = 0%; *p* = 0.67) (Fig 10). No significant changes of point estimates of weighted mean difference were revealed when sensitivity analyses were undertaken. The overall RR did not change substantially when studies of poor methodological quality or in which experimental groups mixed with epinephrine were omitted, it was 0.77 (95% CI 0.57 to 1.04; *p* = 0.09) or 0.87 (95% CI 0.62 to 1.24; *p* = 0.45) (Table 2). The funnel plot presented a fairly symmetrical shape assuming that substantial publication bias was not present (Begg’s test, *p* = 0.707) (Fig 11).

### Adverse effects

Of 13 included studies, seven studies had mentioned the occurence of adverse effects [5,13,16,21,27,29,31]. In the study of De Andres [5] and Allen [21], urinary retention, nausea and vomiting were observed. However, no significant differences were obtained between the combined group and morphine group with regard to these mild to moderate events. In other four studies, no adverse effects were observed in both of the two groups.

## Discussion

This systematic review and meta-analysis of 13 RCTs was conducted to assess the efficacy and safety of IA morphine in combination with bupivacaine compared with morphine alone. The most important findings of the study were that a combination of these two drugs injected intra-articularly could result in lower pain scores during the immediate period (0-2h) and longer time intervals before the first request for rescue analgesia. For analgesia during the two later periods (2-6h, 6-48h) and number of patients requiring supplementary analgesia, no statistically significant differences were observed. In addition, the incidences of adverse events were similar between the two groups.

The major issue of the present systematic review and meta-analysis was to identify whether a combination of morphine and bupivacaine could provide a superior analgesic efficacy when compared with morphine. The result, an immediately superior analgesia provided by IA a combination of morphine and bupivacaine, was supported by some previous findings [21–26]. It might be explained by the early onset and short duration of action of bupivacaine (nearly 2–4 h) [6,22]. However, there were also some evidences suggesting an opposite result [16,27,31]. Many factors could contribute to the controversial effect of using IA morphine and bupivacaine when compared with morphine alone. Owing to small surgical trauma, patients following knee arthroscopy may sometimes experience mild pain, which could make it difficult to optimize study sensitivity [27,33]. Residual analgesia provided by the administration of opioids perioperatively may also have an impact upon assessments of postoperative analgesia [31]. In addition, the volume of fluid injected into the knee joint space should be taken into account. In several trails, the investigators used a 20-ml volume and failed to observe a significant analgesic efficacy [5,13,27,29,31]. It was inevitable that there would be some leakage of injected substances because of oozing of fluid from the operative incision, which result in a decrease in the amount of test drugs in the joint space [27].

Morphine injected intra-articularly have been demonstrated to be safe. Morphine in clinical trials with different doses, ranging from 1 to 15 mg, showed no significant difference comparing with placebo [34–36]. Besides, in the laboratory studies, morphine did not have a significantly negative impact on viability of chondrocytes in the concentrations tested, making itself to be a good alternative IA analgesic [37,38]. In the present systematic review and meta-analysis, seven included studies had reported the occurence of adverse effects, and no significant difference was detect. This finding is very important, for it demonstrated that morphine plus bupivacaine could provide immediately superior analgesia without increasing adverse effects than morphine during the short-term follow-up. The results of this study were in consistent with some previous trails [10,37,39,40]. On the other hand, other studies may argue that bupivacaine may affect chondrocyte viability. Breu et al. and Chu et al. showed chondrotoxic effects of 0.5% bupivacaine in both vitro and vivo studies [39,41]. However, in the studies of Dragoo et al, morphine injected intra-articularly appeared to be safe [42–44]. Recent review by Piper et al. concluded that continuous infusion of bupivacaine at high concentrations in joints with compromised cartilage should be applied with caution, whereas the risk of a single IA injection of bupivacaine remained unclear and further studies were needed [45].

The present study has several strengths. First, this is the first systematic review and meta-analysis comparing the efficacy and safety of IA morphine in combination with bupivacaine with morphine, which is more powerful than previous RCTs and reviews. Second, all of the included 13 original studies were randomized controlled trials, which increases the comparability between the two groups and reduces the possibility of selection bias. Third, the analgesia efficacy of the two groups were compared with in three different periods, and this could make it better to detect the differences between the treatments. Last but not the least, it provides a comprehensive report of the effects of IA a combination of morphine and bupivacaine compared with morphine alone after knee arthroscopy. Given a wide range of geographical locations, patients backgrounds, baseline illness status and ethnicity of the 13 included trials, our findings might have a certain extent of external validity and could potentially be applied to a broader population.

Several limitations of the present study should also be acknowledged. One major limitation of the study was the relatively low quality of the adverse effect evaluation. Chondrolysis, a potentially severe side effect of bupivacaine, needs a longer follow-up time to observe [45]. However, none of the included studies did this long enough observation. A second limitation was the substantial statistical heterogeneity across trials, even though sensitivity analyses were conducted to explore the possible sources of incongruity. In addition, a variety of types of surgeries may have an influence on the results. However, the type of surgeries was consistent between the two groups and the influence on analgesia was equal. At last, since outcome data provided in some trails were not suitable for pooling for meta-analysis, we finally chose three representative indices for evaluating pain control. In spite of that, the number of studies providing data in two outcome measures, time to first request for rescue analgesia and number of patients requiring supplementary analgesia, is relatively small. Therefore, more high-quality RCTs are needed to verify our results.

## Conclusion

The present study suggested that the administration of single-dose intra-articular morphine plus bupivacaine provided better pain relief during the immediate period (0-2h), and lengthened the time interval before the first request for analgesic rescue without increasing the short-term side effects when compared with morphine alone.

## Supporting Information

S1 File PRISMA 2009 Flow Diagram. (DOC)

S2 File PRISMA 2009 Checklist. (DOC)

S3 File Search strategies. (DOC)
