# Supplementary material for: A Single-Dose Intra-Articular Morphine plus Bupivacaine versus Morphine Alone following Knee Arthroscopy: A Systematic Review and Meta-Analysis
Source: PLoS One. 2015 Oct 16;10(10):e0140512. doi: 10.1371/journal.pone.0140512 (PMC4608597; doi:10.1371/journal.pone.0140512)
Supplement: S1 File — (DOC) [file pone.0140512.s001.doc]

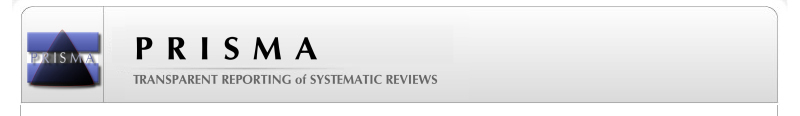
**PRISMA 2009 Flow Diagram**

**Screening**

**Included**

**Eligibility**

**Identification**

Records identified through database searching
(n = 511)

Additional records identified through other sources
(n = 0)

Records after duplicates removed
(n = 239)

Records screened
(n = 239)

Records excluded
(n = 198)

Full-text articles assessed for eligibility
(n = 41)

Full-text articles excluded, with reasons
(n = 28)

Studies included in qualitative synthesis
(n = 13)

Studies included in quantitative synthesis (meta-analysis)
(n = 11)
