# Supplementary material for: A Single-Dose Intra-Articular Morphine plus Bupivacaine versus Morphine Alone following Knee Arthroscopy: A Systematic Review and Meta-Analysis
Source: PLoS One. 2015 Oct 16;10(10):e0140512. doi: 10.1371/journal.pone.0140512 (PMC4608597; doi:10.1371/journal.pone.0140512)
Supplement: S3 File — (DOC) [file pone.0140512.s003.doc]

Supplementary Appendix

Search strategies

PubMed

1. Arthroscopy [tiab] or arthroscopic [tiab] or arthroscope [tiab] or arthrosp* [tiab]
2. Arthroscopy [Mesh]
3. “Anterior cruciate ligament”[tiab]
4. “acl”[tiab]
5. OR/1-4
6. Morphine [Mesh]
7. opiate*[tiab] or opioid*[tiab] or endorphin* [tiab] or morphin*[tiab]
8. OR/6-7
9. Bupivacaine [Mesh]
10. Bupivacain* [tiab]
11. OR/9-10
12. randomized[tiab]
13. placebo[tiab]
14. controlled[tiab]
15. random*[tiab]
16. trial*[tiab]
17. groups[tiab]
18. ((singl*[tiab] or doubl*[tiab] or tripl*[tiab]) and (mask*[tiab] or blind*[tiab]))
19. OR/12-18
20. 5 AND 8 AND 11 AND 19

Embase

1. (Arthroscopy or arthroscopic or arthroscope or arthrosp*):ti,ab
2. ‘Anterior cruciate ligament’/exp
3. (Anterior cruciate ligament):ti,ab
4. OR/1-3
5. ‘Morphine’/exp
6. (opiate* or opioid* or endorphin* or morphin*):ti,ab
7. OR/5-6
8. ‘Bupivacaine’/exp
9. Bupivacain*:ti,ab
10. OR/8-9
11. (random* or control* or trial* or placebo):ti,ab
12. Groups:ti,ab
13. ((singl* or doubl*or tripl*) and (mask* or blind*)):ti,ab
14. OR/8-10

4 AND 7 AND 10 AND 14

Cochrane library

1. MeSH descriptor Arthroscopy explode all trees
2. (Arthroscopy or arthroscopic or arthroscope or arthrosp*):ti,ab,kw
3. MeSH descriptor ‘Anterior cruciate ligament’ explode all trees
4. (Anterior cruciate ligament):ti,ab,kw
5. OR/1-4
6. MeSH descriptor morphine explode all trees
7. (opiate* or opioid* or endorphin* or morphin*):ti,ab,kw
8. OR/6-7
9. MeSH descriptor Bupivacaine explode all trees
10. Bupivacain*:ti,ab
11. OR/9-10
12. 5 AND 8 AND 11
